# The Author Replies

**DOI:** 10.1007/s10654-022-00854-9

**Published:** 2022-03-04

**Authors:** Tamás Ferenci

**Affiliations:** 1 Óbudai Egyetem: Obudai Egyetem, Budapest, Hungary; 2 Department of Statistics, Corvinus University of Budapest, Budapest, Hungary

**Keywords:** COVID-19, Years of life lost, Multimorbidity, Life table, Disease burden, Mortality

I read the letter of Wyper et al. with great interest, and I welcome their excellent contribution to the discussion on years of life lost (YLL) in COVID-19 epidemic.

As emphasized by the title of my paper [1], my aim was to demonstrate the possible approaches to calculate YLL and not to take a strong stance on their merits or applicability, so I very much appreciate every opinion on the strengths and weaknesses of the possible calculation methods. Here, I present a few comments on their letter, and some extensions to my original paper based on their instructive remarks.

First, Wyper et al. state that “[f]rom the perspective of informing public health policy, the counterfactual to be applied in the estimation of YLL is that of an ideal, aspirational, standard based upon desirably low mortality risks” Whether this is indeed true is a matter of debate that I can’t comment on, but Wyper et al. are entirely correct that I failed to present this approach despite being an established way of calculation. I use this opportunity to make up for this omission.

Aspirational calculations either use a fixed target age (e.g., 75 years) to which the years lost are compared, or use a complete life table with “ideally low” mortalities (aspirational life table) [2]. Table 1 presents results for Hungary as of January 31, 2022 using 70, 75, 80 and 85 years target age for the fixed target age method and the Global Burden of Disease (GBD) 2019 Reference Life Table—also known as Theoretical Minimum Risk Life Table—as an aspirational life table (with linear interpolation) [3] as compared to the two metrics presented in my original paper. Note that GBD Reference Life Table has a life expectancy at birth of 88.9 years.

**Table 1:** Years of life lost due to COVID-19 in Hungary with different calculation methods as of January 31, 2022

Method	Years of life lost	Years of life lost per death
Actual Hungarian life table	423,264	10.7
Comorbidity adjustment according to Ferenci, 2021 [1]	370,820	9.38
Aspirational with fixed target age of 70 years	94,058	2.38
Aspirational with fixed target age of 75 years	172,642	4.37
Aspirational with fixed target age of 80 years	282,353	7.14
Aspirational with fixed target age of 85 years	425,594	10.8
Aspirational with the GBD Reference Life Table	708,695	17.9

The calculated YLL with the fixed target age method is almost perfectly quadratic in the target age: $\widehat{YLL} = 16.8 - 0.594 \cdot TargetAge + 0.00526 \cdot TargetAge^{2}$ has an *R*^*2*^ of 99.9%. (Note that for consistency, this includes only deaths above 50 years of age.)

With the exception of the comorbidity-adjusted value, where input data are only available for Hungary, this method can be extended to other countries using the COVarAGE-DB database [4]. Figure 1 shows the results (national life tables for the standard life table method were obtained from the Human Mortality Database; the latest available was used [5]). Here, all data is used, not only those above age 50, as the comorbidity-adjusted value, which would have required this, is not used in this analysis.

**Fig. 1:**
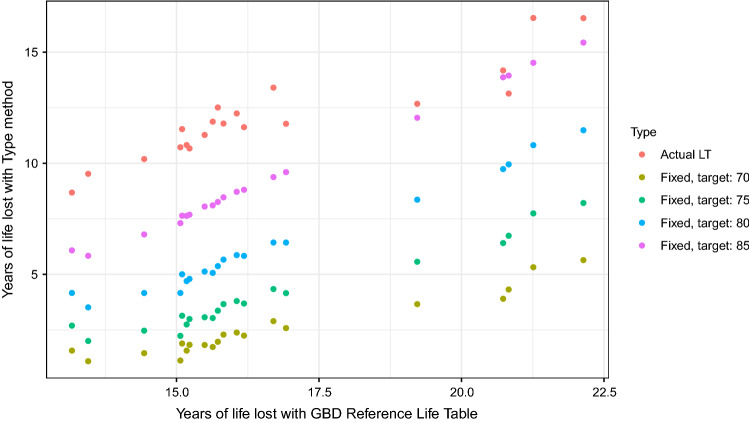
Years of life lost due to the COVID-19 pandemic for 20 countries of the world with different calculation methods: aspirational using the GBD Reference Life Table is on the horizontal axis, other approaches are indicated by the colour (aspirational with fixed target age and actual life table of the country)

Second, they state that “the key utility of YLL estimates lays in comparisons, whether with respect to other health outcomes, across time, or between demographic sub-populations or geographic regions” and claim that correction for comorbidities would likely make such comparisons impossible or infeasible. Indeed, data on individual level comorbidities—that I had for Hungary—are very likely not available for other countries, especially with a uniform methodology in collection (as already noted in the previous point). However, comparison across time is entirely possible, i.e., we could compare different phases of the epidemic within Hungary by calculating the daily number of years of life lost, using uniformly the same adjustment. Not only possible, but it may be also relevant as a way to measure what population is affected by the pandemic (Fig. 2). For instance, if the fatality rate increases in younger age groups, but proportionally decreases at higher ages, it would not be detected by the number of deaths, but plotting YLLs per day picks up this signal. (Wyper et al. states that “any proposals for adjustment would also need to be considered from the alternative perspective, that being that a non-COVID-19 death could be causally related to a prior COVID-19 infection” which is true, but unlikely to be of different magnitude at different points in time, so does not bias such comparisons.)

**Fig. 2:**
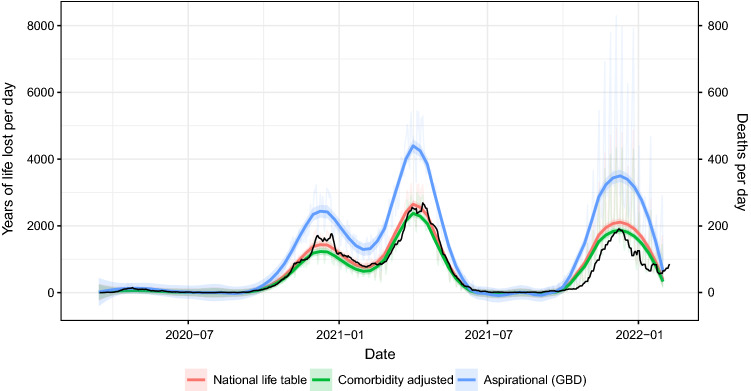
Year of life lost per day (coloured lines, left vertical axis) and number of deaths per day (black line, right vertical axis) due to COVID-19 pandemic in Hungary with smoothing. Non-smoothed value is shown in the background for years of life lost

As an illustration, I calculated the—spline-smoothed—daily number of years of life lost for Hungary with the standard life table method, with the comorbidity-adjusted method, and with the aspirational approach (using the GBD Reference Life Table). One can observe that different results are well aligned, but not perfectly.

Finally, I can wholeheartedly join on their last remark, warning users of such statistics to avoid pitfalls that could lead to unfair and unjust decisions at population level.

My current letter is brief and contains almost no discussion of the presented results, but I hope that it can raise issues that could be fruitfully debated, discussed and extended in the future. Full analysis script (allowing the reproduction of every results presented here) is available at https://github.com/tamas-ferenci/YLL_COVID19_Hungary.

## Data Availability

https://github.com/tamas-ferenci/YLL_COVID19_Hungary.

## Code availability

https://github.com/tamas-ferenci/YLL_COVID19_Hungary.
